# Enhanced controlled drug delivery of berberine-loaded gelatin nanoparticles: characterization and *in vitro* assessment

**DOI:** 10.1039/d5ra08567e

**Published:** 2026-01-29

**Authors:** Hoda A. Sharaf, Mohamed A. Abu Saied, Doaa A. Ghareeb, Sherif H. Kandil, Ahmed Abd El-Fattah

**Affiliations:** a Department of Materials Science, Institute of Graduate Studies and Research, Alexandria University El-Shatby Alexandria 21526 Egypt a_abdelfattah@alexu.edu.eg hoda.sharaf@alexu.edu.eg; b Polymeric Materials Research Department, Advanced Technology and New Materials Research Institute (ATNMRI), City of Scientific Research and Technological Applications (SRTA-City) New Borg El-Arab City 21934 Alexandria Egypt; c Faculty of Industrial and Energy Technology, Borg El-Arab Technological University New Borg El-Arab City 21934 Alexandria Egypt; d Biological Screening and Preclinical Trial Laboratory, Biochemistry Department, Faculty of Science, Alexandria University El-Shatby Alexandria 21526 Egypt; e Pharmaceutical and Fermentation Industries Development Centre, General Authority of City of Scientific Research and Technology Applications Borg El-Arab Alexandria Egypt; f Department of Chemistry, College of Science, University of Bahrain Sakhir P.O. Box 32038 Bahrain

## Abstract

Berberine (BBR), a natural isoquinoline alkaloid, has long been recognized for its potent antimicrobial properties. However, BBR's therapeutic potential remains limited due to its poor bioavailability, low solubility, and short biological half-life. Nanoencapsulation of BBR within a suitable carrier system represents a promising strategy to overcome these limitations and enhance its pharmacological performance. In this study, BBR-loaded gelatin nanoparticles (BBR-GNPs) were successfully synthesized using a double desolvation technique to achieve controlled release and improved antimicrobial efficacy. FTIR and XRD spectroscopy affirmed the loading of BBR in GNPs. The prepared BBR-GNPs exhibited a mean particle size of 215.4 ± 54.32 nm and a zeta potential of +22.6 ± 4.48 mV, as determined by dynamic light scattering (DLS), with an encapsulation efficiency of 72.5%. Scanning electron microscopy (SEM) and transmission electron microscopy (TEM) analyses revealed uniformly spherical nanoparticles with an average size of 169.4 ± 25.89 nm. *In vitro* release studies demonstrated a biphasic and sustained release profile extending over three weeks. The biological evaluation of BBR-GNPs indicated notable antimicrobial, antioxidant, and biocompatibility characteristics. Moreover, MTT assay results showed high cell viability of human skin fibroblasts in a dose-dependent manner, confirming the safety of the developed formulation. Overall, this work presents BBR-GNPs as a promising nanoplatform for controlled drug delivery, offering enhanced solubility, prolonged release, and improved bioavailability of BBR, thereby extending its potential therapeutic applications.

## Abstract 2

Gelatin nanoparticles improve berberine solubility and bioavailability, enabling controlled, sustained release with enhanced antimicrobial, antioxidant, and biocompatible performance, validated *in vitro*.

## Graphical abstract

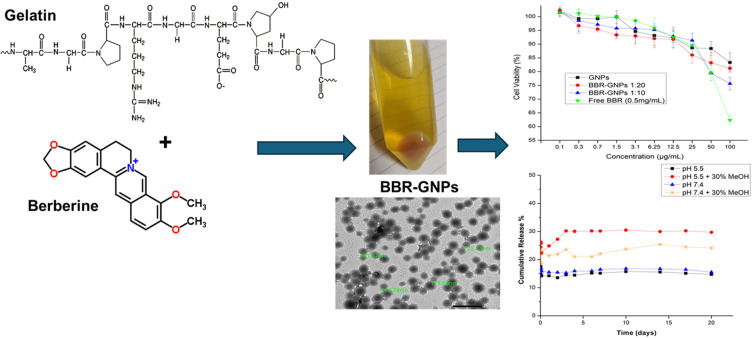

## 1. Introduction

Nanotechnology has attracted immense attention in recent decades due to its rapidly expanding applications across multiple scientific and industrial fields. Among these, nanomedicine stands out as one of the most transformative domains, encompassing nanopharmaceuticals, nanoimaging agents, and theranostic platforms that integrate diagnosis and therapy within a single system.^1^ The use of nanomaterials in medicine offers unprecedented advantages in drug targeting, sustained release, and minimizing systemic toxicity.

Polymeric nanoparticles (PNPs) are particularly appealing nanocarriers owing to their biocompatibility, biodegradability, and tunable physicochemical properties. Typically ranging in size from 10 to 1000 nm, PNPs can encapsulate, entrap, or conjugate therapeutic molecules within a polymeric matrix, thereby improving drug stability and pharmacokinetics.^2^ Biodegradable polymeric biomaterials have gained significant interest as they can be engineered into three-dimensional porous structures, enabling diverse biomedical applications such as tissue engineering, gene delivery, regenerative medicine, and controlled drug delivery systems.^3^

Among various natural polymers, gelatin has emerged as a versatile biopolymer with numerous biomedical applications owing to its low cost, abundance, biocompatibility, and enzymatic degradability. Chemically, gelatin is a denatured collagen-derived polyampholyte that contains both anionic and cationic functional groups, as well as hydrophobic domains, features that facilitate chemical modification, cross-linking, and drug conjugation.^4^ Gelatin nanoparticles (GNPs) have been extensively explored as carriers for drug and gene delivery because of their favorable biological profile and structural adaptability. Since the first report of GNPs in 1978, multiple synthesis strategies have been developed, including single and double desolvation, coacervation-phase separation, emulsification-solvent evaporation, nanoprecipitation, and self-assembly techniques.^5^ The double-desolvation technique was developed as an optimized approach for producing GNPs with controlled particle size, high stability, and reduced aggregation potential. This method has since become the preferred route for fabricating high-quality GNPs suitable for biomedical use.^6^

Berberine (BBR) is a naturally occurring isoquinoline alkaloid found in the roots, rhizomes, and bark of various medicinal plants.^13^ It exhibits a broad spectrum of pharmacological properties, including antibacterial, anti-inflammatory, antioxidant, and anticancer activities.^7,8^ Despite these promising biological effects, the clinical utilization of BBR is severely hampered by its poor aqueous solubility, which arises from its quaternary ammonium structure. Consequently, BBR suffers from low gastrointestinal absorption, limited bioavailability, and a short biological half-life, all of which restrict its therapeutic application and commercial development as a pharmaceutical agent.^9,10^ Moreover, drugs with poor solubility often fail to reach effective plasma concentrations and may induce undesirable side effects when administered intramuscularly or intravenously, including anaphylactic reactions and drug-related rashes.^11,12^

Several polymeric and lipid-based nanocarrier strategies have been explored to overcome BBR's solubility and bioavailability limitations. Lipid-based systems such as solid lipid nanoparticles and nanostructured lipid carriers have been shown to enhance intestinal absorption and improve the pharmacokinetic and hypoglycemic effects of BBR *in vivo*.^13,14^ Poly(lactic-*co*-glycolic acid), PLGA, and poly(ethylene glycol), PEG, formulations, often prepared by nanoprecipitation or emulsion methods, achieved high encapsulation efficiencies and markedly enhanced oral bioavailability and tissue targeting.^15,16^ Additionally, polymeric micelles and functionalized polymer systems (*e.g.*, thiolated Pluronic F_127_ or surface-modified PLGA and chitosan nanoparticles) have been employed to increase skin retention, tumor targeting, and controlled release, yielding superior *in vitro* and *in vivo* efficacy.^17,18^ Collectively, these studies confirm that nanoformulation of BBR substantially enhances its solubility, stability, pharmacokinetics, and therapeutic performance across various biological models.

Despite these promising advancements, most reported BBR-loaded nanocarriers have relied on synthetic or semi-synthetic polymers, which may present biocompatibility issues, residual solvent toxicity, or complex fabrication procedures. Furthermore, only a few studies have focused on natural biopolymer-based carriers such as gelatin, particularly using the double-desolvation approach that allows precise control over particle size and structural stability. In addition, the long-term release behavior and cytocompatibility of BBR-loaded GNPs (BBR-GNPs) have not yet been comprehensively explored.

Therefore, this study aims to develop and characterize BBR-GNPs using a controlled double-desolvation method. The prepared nanocarrier system was designed to enhance the solubility, bioavailability, and retention time of BBR, while achieving sustained and biocompatible drug release. The physicochemical characteristics, *in vitro* release profile, and cytocompatibility of the BBR-GNPs were systematically evaluated to establish a simple, eco-friendly, and effective delivery platform for improving the therapeutic potential of BBR.

## 2. Materials and methods

### 2.1. Materials

Type B gelatin from bovine skin (catalog number: G9382, ∼225 g Bloom; protein content >70%, and molecular weight: 50 kDa), 2,2-diphenyl-1-picrylhydrazyl (DPPH), ethylenediaminetetraacetic acid disodium salt, hydrochloric acid, and potassium hydroxide pellets were purchased from Sigma-Aldrich (St. Louis, MO, USA). Berberine chloride hydrate (97% purity, water content <17%) was obtained from Alfa Aesar (Thermo Fisher Scientific, Karlsruhe, Germany). VISKING dialysis tubing (MWCO 12 000–14 000 Da) was sourced from Heidelberg, Germany. Acetone and Tween 80 were obtained from Piochem (Cairo, Egypt). Anhydrous diammonium hydrogen orthophosphate, disodium hydrogen orthophosphate, dipotassium hydrogen phosphate, glycine (98.5% purity), potassium chloride, and sodium chloride were supplied by Oxford Lab Chem (Maharashtra, India). Dimethyl sulfoxide (DMSO), glutaraldehyde (GLA, 25% aqueous solution), and potassium dihydrogen phosphate were obtained from Loba Chemie Pvt. Ltd (Mumbai, India). Dulbecco's Modified Eagle Medium (DMEM) and fetal bovine serum (FBS) were purchased from Lonza (Basel, Switzerland). Finally, 3-(4,5-dimethylthiazol-2-yl)-2,5-diphenyltetrazolium bromide (MTT) was obtained from Sigma-Aldrich (St. Louis, MO, USA).

### 2.2. Preparation of GNPs and BBR-GNPs

**Fig. 1:**
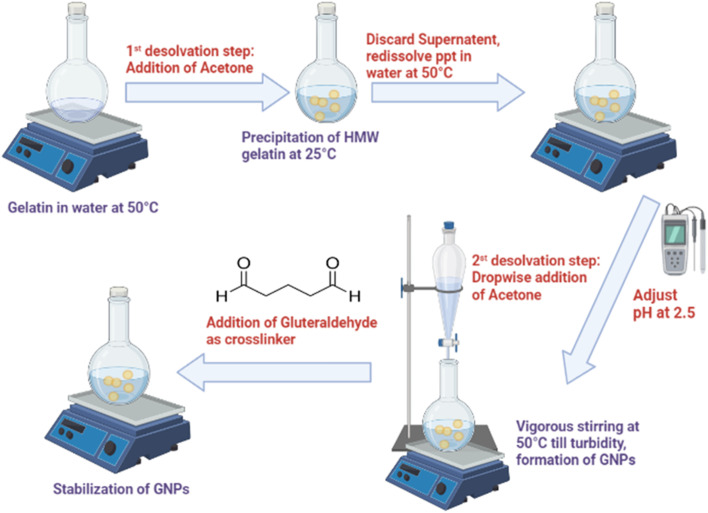
Two-step desolvation method of gelatin nanoparticles (GNPs) synthesis.

GNPs were prepared *via* a two-step desolvation method Fig. 1 as described by Coester *et al.*, with slight modifications.^12^ Briefly, gelatin type B (2 and 5% w/v) was dissolved in Milli-Q water (25 mL) at 50 °C under constant mild stirring until fully dissolved. A volume of cold acetone (25 mL) was added to the gelatin solution as a desolvating agent to precipitate high-molecular weight gelatin, and the supernatant was discarded at room temperature. The precipitated gelatin was redissolved by adding 25 mL Milli-Q water and then stirred at 600 rpm and 50 °C for 1 h. Gelatin solution was adjusted to different pH values *via* acid–base titration. The dissolved gelatin solution was then desolvated again by adding acetone (75 mL) dropwise at a rate of 1 mL min^−1^ under vigorous stirring at 50 °C. To harden the NPs, various amounts of GLA were added, and the solution was incubated overnight at room temperature under stirring at 600 rpm. After that, the glycine solution (7.51 mg mL^−1^) was added and stirred for 1 h to block the residual aldehyde groups (CHO) of GLA. The GNPs solution was subjected to rotary vacuum evaporator to remove the acetone. The dispersion was subsequently centrifuged at 14 000 rpm and 4 °C for 20 min (Histam Plus-Rh centrifuge, 30 065×*g*, Spain). The particles were purified *via* three intervals of centrifugation and vortexed so that they could be redispersed in Milli-Q water. Finally, the resulting NPs were stored at 2–8 °C for further experiments. In this study, we examined the effects of two variables, pH and the amount of cross-linker (GLA), on the NPs production and encapsulation efficiency (EE) of the loaded GNPs. The loading of BBR was performed on the optimized formulation. The loading was implemented *via* the encapsulation technique through the addition of BBR before the second desolvation step. BBR was subsequently dissolved in Milli-Q water containing a gelatin solution at a ratio of 1 : 20. After the addition of a crosslinker and overnight stirring, a yellowish-white colloidal dispersion was obtained. The BBR-GNPs were separated *via* centrifugation at 14 000 rpm and 4 °C for 20 min.

### 2.3. Determination of encapsulation efficiency (EE) and drug loading

The amount of drug encapsulated in the GNPs is equal to the difference between the total amount of the BBR base added in the NPs preparation and the amount of unentrapped BBR base remaining in the clear supernatant after the centrifugation process. The concentration of BBR in the supernatant was measured spectrophotometrically *via* UV-vis (Evolution 300, Thermo Scientific, USA) at 344 nm to obtain the drug concentration after proper water dilution.

The EE was calculated *via*eqn (1):1
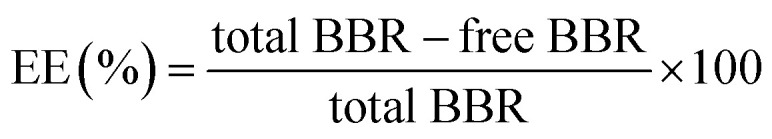


The actual dug content was calculated *via*eqn (2):^19^2
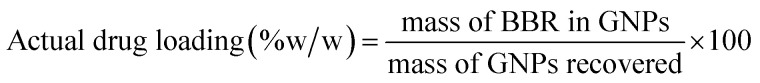


### 2.4. Determination of the practical yield of the prepared GNPs

To determine the amount of GNPs produced *via* the adopted double desolvation method, a volume of purified nanoparticulate suspension was incubated at −80 °C for 24 h and then lyophilized over 72 h at −60 °C until complete dryness and the formation of lyophilized GNPs (Benchtop Freeze Dryer with Omnitronics, USA) were achieved. The percentage yield of the produced NPs was determined with respect to both the weight of the original added amount of gelatin and the weight of the hydrogel, such as the sediment of the gelatin produced in the first desolvation step, which is responsible for producing the homogenous GNPs. Hence, the percentage yield was calculated for the optimized formulation according to eqn (3) and (4):^19^3
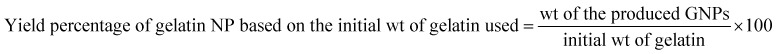
and4
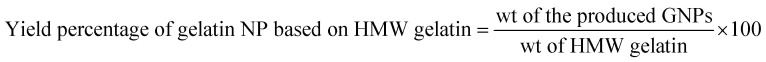


### 2.5. Measurement of particle size, and stability study

The mean particle size (PS) and polydispersity index (PDI) were measured for the prepared NPs using the dynamic light scattering (DLS) technique, and the zeta potential (*ζ*) was measured *via* laser Doppler velocimetry (Zetasizer, Malvern, UK). The samples were prepared by diluting the NP suspension with deionized water and were measured in a 1.0 cm quartz cell at 25 °C. All the measurements were carried out in triplicate (*n* = 3), and the means and standard deviations (SDs) were also calculated. All quantitative data were subjected to statistical analysis as described in Section 2.13.

Long-term stability of plain GNPs and BBR-GNPs was evaluated using lyophilized samples stored under refrigerated conditions (4 ± 2 °C) for five years. The nanoparticles were stored in sealed containers and protected from light throughout the storage period. For analysis, lyophilized samples were reconstituted with distilled water to their original volume and gently vortexed to obtain homogeneous dispersions. The physical stability of the nanoparticles was assessed by measuring the hydrodynamic PS, PDI, and zeta potential using DLS. All measurements were performed in triplicate at 25 °C.

### 2.6. Morphological studies of the prepared nanoparticles

Morphological examination of the nanospheres was carried out *via* a scanning electron microscope (SEM) operated at 20 KeV (JEOL 5300-JSM, Japan). One drop of the nanospheres was placed on a glass slide and well dried under low pressure. It was then coated with gold up to a thickness of 400 Å in a sputter-coating unit prior to imaging by SEM (JEOL Fine Coat Ion Sputter, JFC-1100E, Japan).

The morphology and particle size of the GNPs and BBR-GNPs were analyzed *via* transmission electron microscope (TEM) at accelerating voltages of 80 kV and 25 °C (JEOL 1400 PLUS, Japan). A drop from the prepared sonicated formulations was placed on a carbon film-covered copper grid (200-mesh) with 2% (W/V) uranyl acetate stain.^20^ The grid was subsequently air-dried at room temperature before being inserted into the microscope.

### 2.7. Fourier transform infrared spectroscopy (FTIR) analysis

FTIR spectra were recorded *via* a UV-visible spectrometer (FTIR-8400S, Shimadzu, Japan) throughout the range from 4000 cm^−1^ to 400 cm^−1^ for spectroscopic identification of the functional groups present in the gelatin powder, BBR powder, GNPs, and BBR-GNPs. The samples were prepared as KBr discs (2 mg sample in 200 mg KBr) with a hydrostatic press at a force of 40 psi for 4 min.

### 2.8. X-ray diffraction (XRD) analysis

XRD was performed using a diffractometer with Cu Kα radiation (*λ* = 0.1542 nm) at an operating voltage of 40 kV (XRD-7000, Shimadzu, Kyoto, Japan). The patterns were recorded over a Bragg angle (2*θ*) range of 10–80° for BBR powder, GNPs and BBR-GNPs.

### 2.9. Antimicrobial activity

The antimicrobial activity of all the samples was determined *via* a turbidity assay.^21^ Three microbial species known to be pathogenic, namely, Gram-negative bacteria (*E. coli* ATCC25922), Gram-positive bacteria (*Staphylococcus aureus* ATCC25923) and yeast (*Candida albicans* EMCC105), were tested. The strains were grown in nutrient broth at 37 °C for 24 h. Broth cultures of bacteria were suspended overnight in Mueller–Hinton (MH) broth with the turbidity adjusted to 0.5 McFarland, resulting in a suspension containing approximately 1 × 10^8^ CFU mL^−1^. To measure the minimum inhibitory concentration (MIC), the MH broth culture mixture (50 µL) was poured into 12 wells of a 96-well microtest plate in accordance with the Clinical and Laboratory Standards Institute guidelines.^22^ In the first well, the sample stock solution (50 µL) was added. A 2-fold dilution was then made to obtain different concentrations of samples in each well (µg mL^−1^). The microbial suspension (50 µL) was subsequently added to each well. The microplate was then incubated at 37 °C for 24 h, and the sample concentration in the well without visible growth of the bacterial cells was considered the MIC. A positive control (maximum bacterial growth) contained MH broth medium only with tested bacterial concentration. The test was carried out in comparison with (10 mg mL^−1^) amoxicillin. The optical density was measured at 600 nm. The MIC was defined as the least concentration of sample that visually inhibited bacterial growth after 24 h of incubation. The MIC was determined by observing the visual turbidity of the wells before and after incubation. Pathogenic strain inhibition (%) was calculated as follows:^23^5
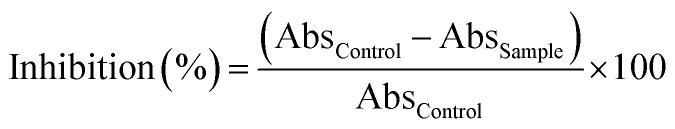


### 2.10. *In vitro* drug release studies

GNPs incorporating equivalent concentrations of BBR (1 mg) were entrapped in a cellulose dialysis membrane and dialyzed against (40 mL) PBS media with four modifications according to pH (5.5, 7.4) with or without 30% methanol at 37 °C and 100 rpm in a shaker incubator (LSI-3016A, Labtech, South Korea). At predetermined time intervals, and 2 mL sample were withdrawn from the dissolution medium and replaced with fresh medium to ensure sink conditions.^24^ The cumulative amount of drug released was spectrophotometrically measured at 344 nm over 21 days. The inclusion of 30% methanol was utilized to ensure sink conditions for the poorly water-soluble BBR, allowing for accurate characterization of the intrinsic release kinetics by preventing premature saturation of the medium.

### 2.11. Free radical scavenging activity

The free radical scavenging activity of the nanosystem was measured by the 2,2-diphenyl 1,1-picryl hydrazyl (DPPH) method as proposed by Brand-Williams, with some modifications.^25^ A solution of DPPH (0.2 mM) in methanol was prepared, and 1 mL of the radical solution was added to 1 mL of each sample at different concentrations (1 : 1 v/v) of the tested formula. The mixture was incubated for 30 min in the dark at room temperature. For the control (no radical scavenging activity), 100 µL of the DPPH solution was mixed with buffer instead of the nanosystem solution. The absorbances of the control (Abs_Control_) and samples (Abs_Sample_) were subsequently measured at 517 nm *via* a microplate reader (Accu Reader, Taiwan). Ascorbic acid solution was used as a standard in the concentration range of 5–200 µg mL^−1^ to establish a standard curve. DPPH radical scavenging activity was expressed as mg ascorbic acid equivalent (AAE) per g dried sample. The percentage of DPPH radical-scavenging activity was calculated *via*eqn (6):^26^6
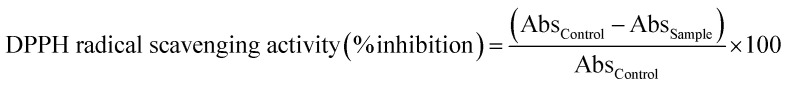


All the determinations were performed in triplicate, and the average values were tabulated.

### 2.12. Cytotoxic effect against human skin fibroblasts (HSFs) assessment

HSFs were obtained from Nawah Scientific, Inc. (Mokatam, Cairo, Egypt). The cells were maintained in DMEM supplemented with 100 mg per mL streptomycin, 100 units per mL penicillin and 10% heat-inactivated fetal bovine serum in a humidified 5% (v/v) CO_2_ atmosphere at 37 °C.

Cell viability was assessed *via* 3-(4,5-dimethylthiazol-2-yl)-2,5-diphenyltetrazolium bromide (MTT) assays. MTT was used to detect mitochondrial metabolism and the viability of the cells. Tetrazolium salt is reduced to insoluble formazan by mitochondrial dehydrogenase.^27^ Aliquots of 100 µL of cell suspension (5 × 10^3^ cells) were seeded in 96-well plates and incubated in complete media for 24 h at 37 °C in 5% CO_2_. The cells were treated with another aliquot of 100 µL media containing drugs at various concentrations. After 48 h of drug exposure, the media was discarded, and MTT solution (20 µL of 1 mg per mL stock solution) was added to 100 µL of PBS in each well and incubated at 37 °C for 4 h. Then, the formed formazan crystals were dissolved in 100 µL of absolute DMSO. The absorbance of the formazan solutions was measured at *λ*_max_ 570 nm *via* a multi-well plate reader (BMG LABTECH®FLUOstar Omega, Germany).7
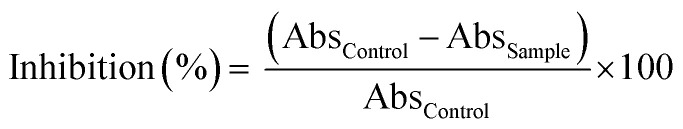


### 2.13. Statistical analysis

The quantitative data are presented as the means ± SDs to analyze the differences among the experimental groups. Statistical analysis was performed using one-way analysis of variance (ANOVA), and a *p*-value ≤ 0.05 was considered statistically significant.

## 3. Results and discussion

### 3.1. Optimized encapsulation and loading parameters of BBR-GNPs

In this study, we assessed the impact of two parameters, pH and the amount of cross-linker (GLA), on the GNPs production and EE of the loaded GNPs. The results revealed that pH 2.5 was the optimum pH for NPs preparation Table S1. It has been claimed that creating enough charges on the gelatin surface causes the development of NPs and prevents subsequent agglomeration.^28^ The process of GNPs development involves regulated precipitation, which is caused by the loss of water molecules and regulated by intermolecular charge repellent forces generated by the altered pH.^29^

Furthermore, as shown in Table S2, the optimum amount and concentration of GLA are 185 µL and 25% v/v, respectively, and longer crosslinking times of 16 h and a steady stirring speed are associated with stable homogenous NPs with a low PDI, the smallest PS, the maximum EE of BBR (72.48% ± 3.27) and the DL (10.62% ± 2.33) of the loaded GNPs. This can be explained by the fact that higher amounts of GLA cause the particles to become more entangled through interaction with the –NH_2_ groups of the gelatin and prevent the particles from swelling in water, which increases the stability and hardens of gelatin nanospheres.^30^

In addition, drug entrapment within the nanosphere matrix is achieved by incorporation method through incorporating the drug before the second desolvation step to bind it physically with the polymer *via* electrostatic interactions and hydrogen bonding. The entrapment of BBR in GNPs could be based on the preferential localization of the drug inside the nanosphere matrix, which is less hydrophilic than the external aqueous environment; furthermore, as desolvation eliminates water from the core, the EE for this drug is increased.

During the fabrication of the GNPs, the temperature was maintained at 50 °C with a low stirring speed to avoid agglomeration and aggregation caused by both thermal and mechanical stresses. Additionally, the acetone addition rate was adjusted to 1 mL min^−1^ because higher rates can result in water elimination from gelatin and aggregation during preparation.^31^

The yield percentage of the prepared GNPs was 34.79 ± 7.4%, which is in accordance with previous reports.^32^ This average yield obtained for the GNPs could be attributed to the discarding of some LMW gelatin chains during the initial desolvation step. Additionally, the yield percentage of HMW gelatin was found to be 50.34 ± 10.35%.^30^

### 3.2. Physicochemical characterization and long-term stability of BBR-GNPs

**Fig. 2:**
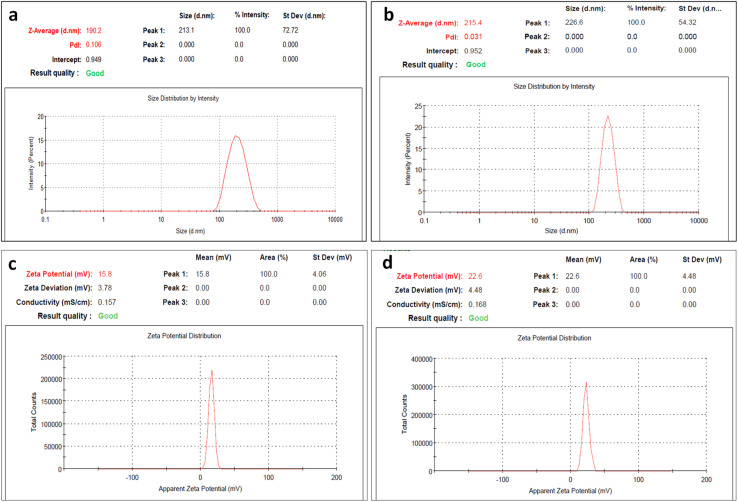
Particle size distribution of (a) GNPs and (b) BBR-GNPs and zeta potential measurements of (c) GNPs and (d) BBR-GNPs.

Both PS and PDI are key factors in the application of nanotechnology for improving drug delivery systems, as they significantly affect the targeting properties, destiny, *in vivo* biodistribution, and toxicity of such nanodelivery systems.^19^ The mean PS distribution of the GNPs measured *via* DLS was 190.2 ± 72.72 nm, which was associated with a PDI value of 0.106, as shown in Fig. 2a, whereas the mean PS of the BBR-loaded GNPs was 215.4 ± 54.32 nm, which was associated with a very low PDI value of 0.031, as shown in Fig. 2b. These data indicate that the GLA reacted with the amino groups on the same particle instead of binding two particles together, resulting in effective and homogeneous incorporation of BBR throughout the matrix of the produced gelatin nanospheres. The PS values implicate that the size of the BBR-GNPs is larger than that of the GNPs; therefore, these results prove that the BBR drug was successfully encapsulated into the GNPs. This could demonstrate the effectiveness of the double desolvation approach in producing uniform PS owing to the elimination of the LMW and intermediate gelatin chains.

The zeta potentials of the GNPs and BBR-GNPs were 12.9 ± 4.75 mV and 22.6 ± 4.48 mV, respectively, as presented in Fig. 2c and d. The surface charge features of these GNPs could be attributed to their corresponding zeta potentials. The relatively high positive zeta potential of the GNPs could be explained by the HMW of the gelatin, resulting in a greater density of amine (NH_3_^+^) groups at the surface, which are acquired during the preparation of the GNPs in acidic solution (pH 2.5). The loading of BBR dramatically raised the net positive charge of the GNPs (*P* < 0.05), indicating a statistically significant difference.

A high absolute zeta potential represents a greater electric charge on the BBR-loaded GNPs surface, which might create strong repelling forces among the particles, preventing aggregation or sedimentation of the GNP solution. As a result, it is a significant index for the stability of the GNP suspension.^33^

The long-term stability of lyophilized GNPs and BBR-GNPs stored at 4 ± 2 °C for five years was evaluated by comparing their physicochemical properties with freshly prepared formulations. As summarized in Table 1, an increase in PS and PDI, accompanied by a reduction in zeta potential, was observed after long-term storage. Such changes are commonly reported for lyophilized polymeric nanoparticles and are primarily attributed to partial aggregation upon reconstitution, polymer chain relaxation, and increased intermolecular hydrogen bonding during prolonged storage, particularly in the absence of cryoprotectants.^34,35^ Despite the increase in particle size, both GNPs and BBR-GNPs remained within the nanoscale range (<500 nm), and no visible aggregation or precipitation was detected, indicating preservation of colloidal integrity.

**Table 1:** Particle Size, PDI, and zeta potential of freshly prepared and long-term stored formulations

Parameter	GNPs	5-year GNPs	BBR-GNPs	5-year BBR-GNPs
Size (nm)	190	492.2	215.4	414
PDI	0.106	0.448	0.031	0.364
Zeta (mV)	15.8	5.48	22.6	10.9

Notably, BBR-GNPs exhibited superior stability compared to plain GNPs after long-term storage, as evidenced by their smaller particle size, lower PDI, and higher zeta potential values. This improved stability may be attributed to intermolecular interactions between berberine and the gelatin matrix, which can restrict polymer mobility and reduce aggregation tendencies.^12,36^ The observed decrease in zeta potential over time is consistent with surface charge shielding and molecular rearrangement phenomena reported for gelatin-based and other polymeric nanoparticle systems during extended storage and does not necessarily indicate formulation failure.^37^ Overall, the results demonstrate that lyophilized BBR-GNPs maintain acceptable physicochemical stability after five years of refrigerated storage, supporting their suitability for long-term storage and subsequent use in localized or controlled drug delivery applications rather than immediate intravenous administration.

### 3.3. Morphological characterization of BBR-GNPs

**Fig. 3:**
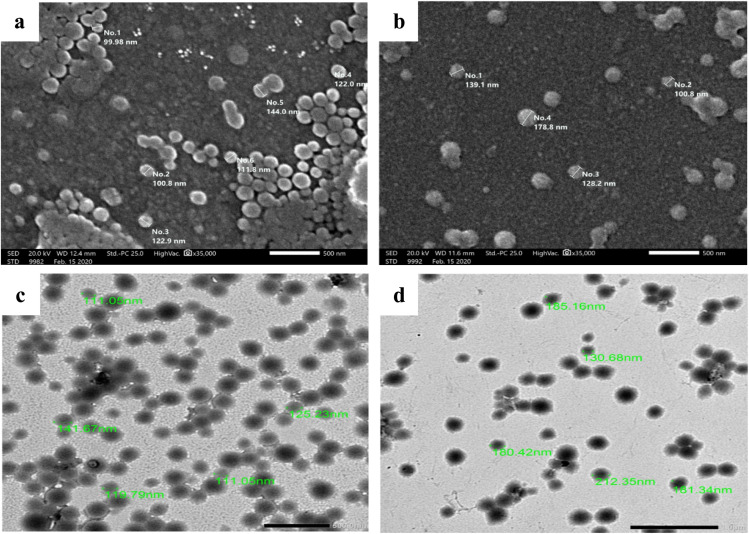
Scanning Electron Microscope (SEM) micrographs: (a) GNPs and (b) BBR-GNPs and Transmission Electron Microscopy (TEM) images (×12 000): (c) GNPs and (d) BBR-GNPs.

The surface morphology of the GNPs and BBR-GNPs was investigated *via* SEM and TEM. The SEM micrographs revealed a smooth spherical shape of the formed NPs. Fig. 3a revealed some aggregates with relatively homogenous sizes of GNPs, which may be due to the sample preparation, which caused dehydration and sample shrinkage of the protein structure, resulting in the formation of NP aggregates. Fig. 3b shows that the larger size of the BBR-GNPs is due to the drug being encapsulated in the gelatin nanospheres.^38^ TEM morphological investigation of the prepared GNPs revealed that the particles were homogeneous and spherical regular vesicles entrapping BBR, which was indicated by the darkness of the core of the observed vesicles, as shown in Fig. 3c and d. The mean PSs of the GNPs and BBR-GNPs were 116.78 ± 6.98 nm and 169.4 ± 25.89 nm, respectively, as shown in the TEM micrographs. Moreover, the size of the TEM images correlates well with the means of the PS observations obtained *via* the particle size measurements. The observed difference between DLS (hydrodynamic diameter) and TEM (dry diameter) measurements is an expected phenomenon, as DLS includes the hydration layer and solvent molecules moving with the particle, while TEM visualizes the dry core.

### 3.4. FTIR spectroscopic confirmation of BBR encapsulation

**Fig. 4:**
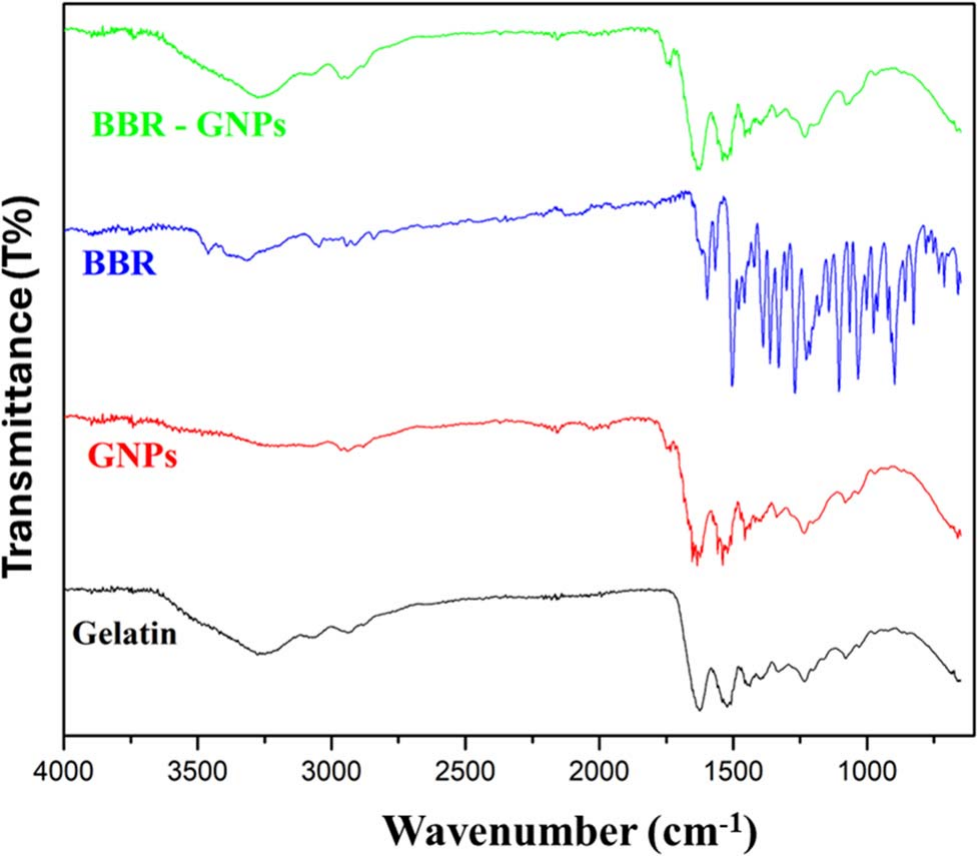
FTIR spectra of gelatin powder, GNPs, BBR powder and BBR-GNPs.

As shown in Fig. 4, the FTIR spectra of all the gelatin, GNPs, BBR, and BBR-GNPs samples present broad –OH stretching and amide (–NH) bands between 3500 and 3100 cm^−1^.^39^ The FTIR spectra of gelatin and GNPs are characterized by amide B asymmetric stretching of CH_2_ groups at 2880 cm^−1^, an amide I peak at 1645 cm^−1^, an amide II peak of N–H stretching and a C–N bending peak at 1552 cm^−1^.^40^ The presence of the same peaks for gelatin and GNPs indicates that there is no chemical interference from any of the reagents during preparation. The spectrum of BBR is characterized by the methoxy group (–OCH_3_) at 2842.8 cm^−1^, aromatic C=C bending at 1597.2 and 1567.5 cm^−1^, aromatic C=C of the furan group at 1504.3 cm^−1^, and C–H vibration at 1033.7 cm^−1^.^41^ The spectrum of BBR-GNPs shows an increase in absorption intensity and a broad band of –OH stretching at (3311.9–3196.1 cm^−1^), where the hydroxyl group is dominant in GNPs and BBR.^38^ The spectra of the GNPs and BBR-GNPs clearly have nearly the same characteristic peaks. In addition, the disappearance of the methyl group peak (2842.8 cm^−1^) from the BBR-GNPs spectrum is attributed to the entrapment of BBR in the host cavity of the nanocarriers.

### 3.5. XRD analysis: crystalline state and amorphization of BBR

**Fig. 5:**
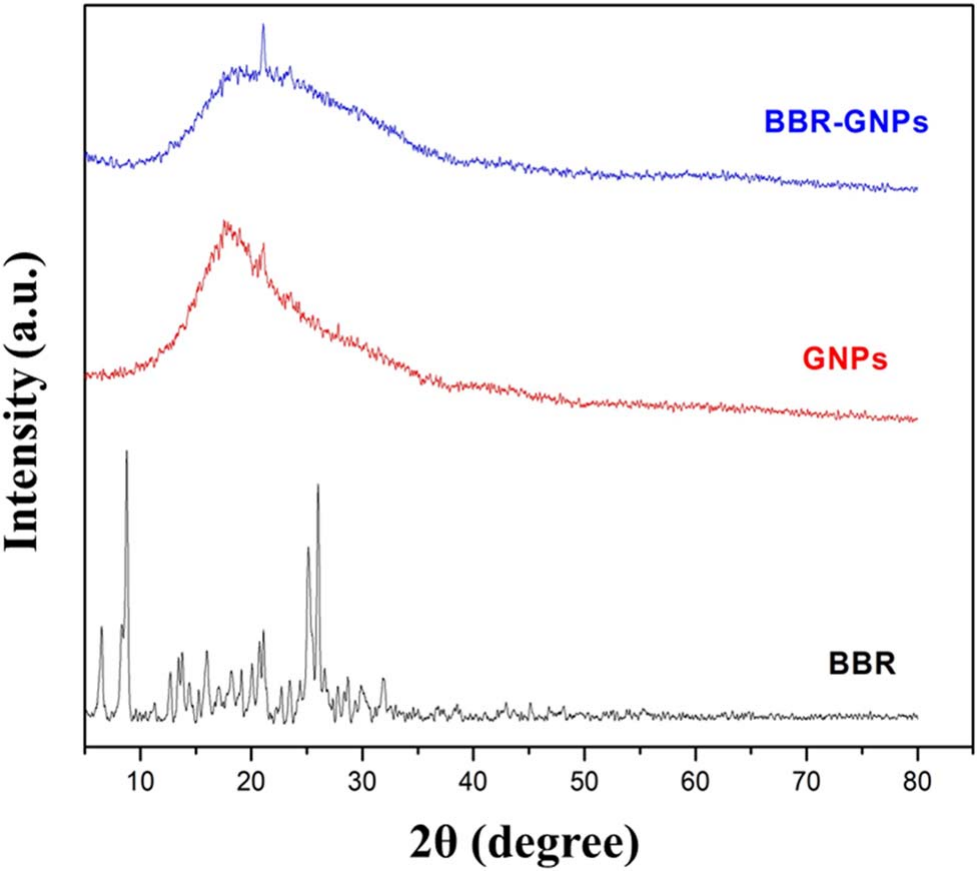
XRD patterns of BBR powder, GNPs and BBR-GNPs.

The XRD patterns of BBR, GNPs, and BBR-GNPs are shown in Fig. 5. The XRD pattern of BBR powder has strong and powerful diffraction peaks at 2*θ* values of 8.8°, 16°, 20.7°, and 25.1°, which indicate that BBR is crystalline in nature.^22^ The XRD patterns of the GNPs exhibit a sharp peak at 21.1°, which indicates the restructuring of the collagen as a triple helix structure and the semicrystalline nature of the material. The diffraction pattern of the prepared BBR-GNPs exhibited a broad peak of decreasing intensity, indicating that the crystallinity of the drug decreased, and that the crystalline nature of the material changed to an amorphous form.^42^ Compared with their crystalline forms, amorphous materials have more free energy. As a result, less crystalline or amorphous forms of drugs are more easily soluble and dissolve at a higher rate than their crystalline counterparts are. Thus, modifying the crystalline structure through nanosizing may be an effective strategy for increasing drug molecule solubility and dissolution rates, further enhancing bioavailability.^22^

### 3.6. *In vitro* antimicrobial efficacy of BBR-GNPs

BBR is the main antibacterial component in the prepared nanosystem and has broad-spectrum antibacterial action, particularly against Gram-positive bacteria such as *Staphylococcus aureus*. In the present study, which used a turbidity assay, the GNPs did not show antimicrobial activity against the tested pathogenic strains because gelatin is a protein biopolymer. According to Table 2, the formulations containing 2% BBR-GNPs and 5% BBR-GNPs had moderate inhibitory effects on all the tested pathogenic strains, in contrast to the significantly high inhibitory effects of the unprocessed BBR and amoxicillin antibiotics. Consequently, as the BBR concentration loaded on the GNPs increased, the minimum inhibitory concentration (MIC) increased, so the percent inhibition increased. These findings prove that the active component (BBR) is responsible for antibacterial activity, where the BBR released from the nanospheres inhibits the active transport and respiration of bacteria, resulting in growth suppression and death.^43^

**Table 2:** Antimicrobial activity using turbidity assay

Pathogenic strain tested material	Inhibition (%)	Sample concentration (µg mL^−1^)
**Gram-negative bacteria *E. coli* ATCC25922**		
BBR-GNPs 2%	11.11	16.03
BBR-GNPs 5%	35.65	30.12
BBR	60.06	50
GNPs 2%	ND	50
Amoxicillin	77.51	0.01

**Gram-positive bacteria *Staphylococcus aureus* ATCC25923**		
BBR-GNPs 2%	13.70	16.03
BBR-GNPs 5%	15.78	30.12
BBR	79.61	50
GNPs 2%	ND	50
Amoxicillin	90.43	0.01

**Fungi *Candida albicans* EMCC105**		
BBR-GNPs 2%	7.17	16.03
BBR-GNPs 5%	14.79	30.12
BBR	38.06	50
GNPs 2%	ND	50
Amoxicillin	43.75	0.01

The antimicrobial activity of BBR is pleiotropic. Unlike amoxicillin, which targets cell wall synthesis and may exhibit higher *in vitro* potency at lower concentrations, BBR primarily disrupts the bacterial cell membrane and inhibits efflux pumps. Besides, BBR increases the permeability of the bacterial plasma membrane, leading to the leakage of intracellular contents (K^+^ ions, proteins, and DNA). Furthermore, BBR is a known inhibitor of the FtsZ protein, which is essential for bacterial cell division.^44^ This pleiotropic mechanism inherently makes BBR less prone to resistance development compared to single-target antibiotics, offering a significant advantage in combating antimicrobial resistance. GNPs, being protein-based biopolymers, exhibit high bioadhesion. This allows the BBR-GNPs to maintain prolonged contact with the bacterial cell wall, facilitating a localized high concentration of BBR that enhances the disruption of the membrane more effectively than the rapidly cleared free BBR.^45^ This nanoencapsulation strategy effectively addresses BBR's poor bioavailability and short half-life, transforming it into a more viable therapeutic agent. BBR has also been shown to induce the production of Reactive Oxygen Species (ROS) within the bacterial cytoplasm. This oxidative stress leads to lipid peroxidation of the membrane and irreversible damage to bacterial DNA.^46^ Coupled with the inherent biocompatibility of gelatin and the controlled, sustained release profile of BBR-GNPs, this formulation presents a safer and more durable therapeutic strategy, particularly relevant for localized, prolonged drug delivery and in the broader context of rising antimicrobial resistance.

### 3.7. *In vitro* sustained drug release profile

**Fig. 6:**
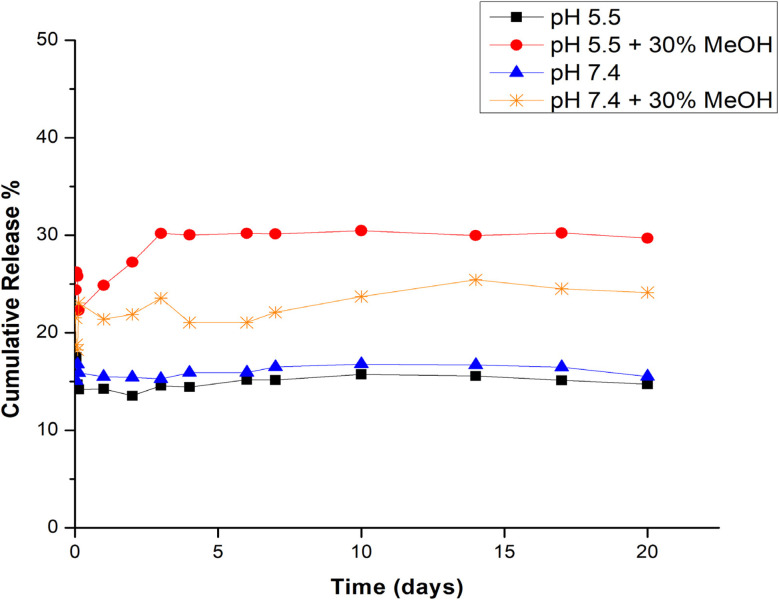
Percentage cumulative release of BBR from GNPs in different PBS media at 37 °C and 100 rpm.

On the basis of the results of the solubility study shown in Fig. S1, the release of BBR from GNPs was carried out in PBS at pH 5.5 and 7.4 with or without 30% methanol at 37 °C and 100 rpm. The cumulative percentage of BBR released over 21 days is shown in Fig. 6. After 10 days of incubation in PBS media at pH 5.5 and 7.4, the cumulative percentage of BBR released from the GNPs was 15.74% and 16.79%, respectively. PBS at pH 7.4 acts as a slightly alkaline medium for the GNPs (isoelectric point ∼5.7), which reduces the surface positive charges due to suppressed protonation of the amino groups and thus obstructs the release of positively charged berberine.^19^ Notably, after 10 days, the addition of 30% MeOH to each buffer at pH 5.5 and 7.4 caused a significant increase in drug release from the GNP matrix of 30.48% and 23.72%, respectively, which indicated that the GNPs released more drug into the acidic medium.

All release patterns are biphasic and display similar release behavior, with an early surge in the first 2 h due to the rapid liberation of surface-bound BBR from the gelatin matrix. This was followed by a steady release of the drug, which was limited by the diffusion of BBR and its poor dissolution from the cross-linked gelatin matrix. Additionally, the inner collapsed core of the gelatin matrix delayed the release of the remaining BBR in a slow, sustained manner. According to a previous study, this behavior was attributed to an increase in the gelling of the polymer hindering the sudden release of the drug.^47^

For controlled drug release, steady behavior can be used to manage drug concentrations below the toxicity threshold and retain the drug within the therapeutic period.^48^ Consequently, the persistent gradual release of BBR from the NPs affirms the applicability of BBR-GNPs in controlled drug release applications.

### 3.8. Antioxidant properties and free radical scavenging

**Fig. 7:**
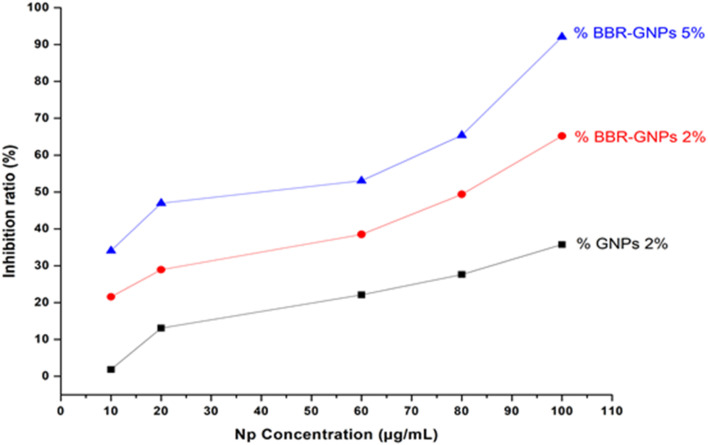
DPPH radical scavenging activities of GNPs (2%) and BBR-GNPs (2% and 5%).

The 2,2-diphenyl 1,1-picryl hydrazyl (DPPH) radical scavenging assay demonstrated the ability of the nanoparticulate samples to transfer electrons or hydrogen atoms. Owing to their scavenging ability, antioxidants can scavenge free radicals and reduce their adverse effects, where many diseases are linked to the accumulation of free radicals.^49^ The DPPH free radical scavenging effect increased with increasing concentrations of the test samples; the percentages of samples enriched with 2% GNPs, 2% BBR-GNPs, and 5% BBR-GNPs were 35.73%, 65.19%, and 92.08%, respectively Table S3. As shown in Fig. 7, the sample containing 5% BBR-GNPs exhibited the highest antioxidant activity and free radical scavenging ability. As a result, the loading of BBR on the nanocarrier resulted in high antioxidant activity, which may be due to size reduction and the exposure of many OH groups.^50^ DPPH was used to examine the antioxidant activity of Nano-BBR in comparison with unprocessed BBR, and it was discovered that Nano-BBR exhibited high antioxidant activity, as reported by Sharifi-Rad *et al.* 2020.^51^ BBR acts as a high-efficiency antioxidant by directly scavenging ROS and indirectly triggering the AMPK pathway, which enhances the synthesis of endogenous enzymes such as SOD and CAT. It provides comprehensive protection against oxidative injury by suppressing ROS formation inmitochondria and NADPH oxidase, coupled with the upregulation of the protective Nrf2 transcription factor.^52^ While direct experimental quantification of ROS for our specific BBR-GNPs was not performed in this study, the observed antioxidant activity is consistent with BBR's well-documented mechanisms.

### 3.9. *In vitro* cytotoxicity and biocompatibility evaluation

An MTT assay was used to assess the viability of the cells subjected to the prepared samples of BBR-GNPs at two different ratios (1 : 20 and 1 : 10) to investigate the impact and biocompatibility of the nanoparticulate systems on normal human skin fibroblasts (HSFs). HSFs were chosen as a relevant cell model for initial biocompatibility assessment due to their common use in *in vitro* safety evaluations.

**Fig. 8:**
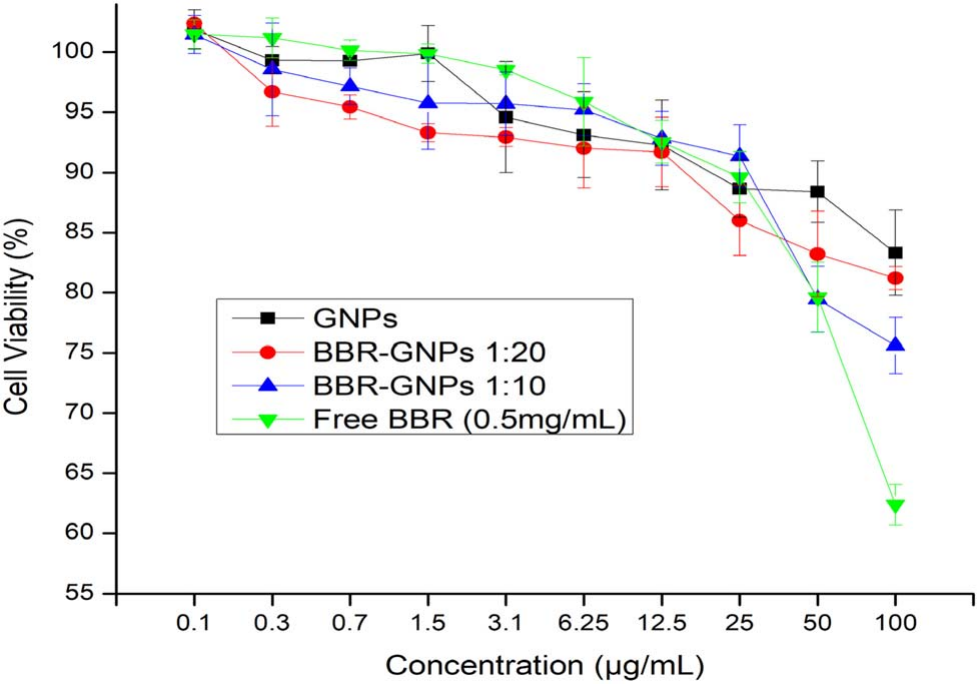
MTT assay shows cell viability of HSF treated with GNPs, BBR-GNPs 1 : 20, BBR-GNPs 1 : 10 and free BBR with different concentrations (0–100 µg mL^−1^) at 48 h. Graph columns values represent mean of viable cells ± S.D.

HSFs were incubated with a wide range of GNPs, BBR-GNPs and BBR concentrations (0–100 µg mL^−1^) for 48 h. Fig. 8 shows the cell viability of HSF as a function of the BBR concentration. The viability of the cells treated with GNPs did not significantly decrease. BBR-GNPs at ratios of 1 : 20 and 1 : 10 presented high viability (81.2% and 75.6%, respectively), even at the highest concentration (100 µg mL^−1^). After treatment of HSFs with free BBR at various concentrations (0–25 µg mL^−1^), cell viability was not significantly affected. Alternatively, exposure to higher doses of free BBR (50 µg mL^−1^ or 100 µg mL^−1^) resulted in lower viability (79.6% and 62.4%, respectively).

In our study, no significant cytotoxicity was observed against normal noncancerous fibroblasts, which implied that the BBR-GNPs drug delivery system has good biocompatibility. Additionally, a hemolysis test was performed to confirm hemocompatibility *via* red blood cells (RBCs). The results for the BBR-GNPs revealed a 3.5-fold reduction in the hemolysis of the RBCs compared with that of the GNPs, as shown in Fig. S2. Therefore, as the IC_50_ increases, the nanoparticulate formula (BBR-GNPs) shows good biocompatibility in hemostatic applications, as shown in Table S4.

## 4. Conclusion

BBR loaded GNPs were successfully prepared using a two-step desolvation method, resulting in stable, uniform nanocarriers with favorable physicochemical characteristics. The optimized formulation showed nanosized particles with a positive surface charge, good homogeneity, and high encapsulation efficiency. The *in vitro* release studies demonstrated a biphasic and sustained release profile that extended for three weeks, confirming the system's ability to maintain prolonged drug delivery. The prepared BBR-GNPs exhibited notable antimicrobial and antioxidant activities together with excellent cytocompatibility toward human skin fibroblasts, indicating their biological safety. Overall, the developed BBR-GNPs provide an efficient and biocompatible platform for improving the solubility, stability, and therapeutic performance of berberine. Building on these promising *in vitro* results, *in vivo* studies are currently being conducted within our research group to thoroughly evaluate their pharmacokinetic profile, biodistribution, and therapeutic efficacy in relevant animal models, thereby paving the way for eventual translational application.

## Conflicts of interest

The authors declare that there is no conflict of interest.

## Supplementary Material

RA-016-D5RA08567E-s001

## Data Availability

All data supporting the results of this article are included in the article and its supplementary information (SI). Supplementary information is available. See DOI: https://doi.org/10.1039/d5ra08567e.
