# Supplementary material for: Enhanced controlled drug delivery of berberine-loaded gelatin nanoparticles: characterization and in vitro assessment
Source: RSC Adv. 2026 Jan 29;16(7):6119–31. doi: 10.1039/d5ra08567e (PMC12853093; doi:10.1039/d5ra08567e)
Supplement: RA-016-D5RA08567E-s001 [file RA-016-D5RA08567E-s001.pdf]

Supplementary Information (SI) for RSC Advances.
This journal is © The Royal Society of Chemistry 2026

Enhanced Controlled Drug Delivery of Berberine-Loaded Gelatin Nanoparticles: Characterization and *in Vitro* Assessment

Hoda A. Sharaf[1*], Mouhamed A. Abu Saied[2,3], Doaa A. Ghareeb[4,5], Sherif H. Kandil[1], Ahmed Abd El-Fattah[1,6*]

[1] Department of Materials Science, Institute of Graduate Studies and Research, Alexandria University, El-Shatby, Alexandria 21526, Egypt.

[2] Polymeric Materials Research Department, Advanced Technology and New Materials Research Institute (ATNMRI), City of Scientific Research and Technological Applications (SRTA-City), New Borg El-Arab City 21934, Alexandria, Egypt.

[3] Faculty of Industrial and Energy Technology, Borg El-Arab Technological University, New Borg El-Arab City 21934, Alexandria, Egypt.

[4] Biological Screening and Preclinical Trial Laboratory, Biochemistry Department, Faculty of Science, Alexandria University, El-Shatby, Alexandria 21526, Egypt.

[5] Pharmaceutical and Fermentation Industries Development Centre, General Authority of City of Scientific Research and Technology Applications, Borg El-Arab, Alexandria, Egypt.

[6] Department of Chemistry, College of Science, University of Bahrain, Sakhir P.O. Box 32038, Bahrain.

Supplementary Material

Optimization of the Prepared Nanoparticle Formulations

Table S1. Different pH values and apparent observations in GNPs preparation

pH value	Condition
2.5	Translucent colloidal dispersion
3.5	Turbid colloidal dispersion
5	Gelatinous precipitate
8	Yellow viscous precipitate
10	Opaque yellow solution
11	Opaque pink solution
12	Gelatinous fragments

The increasing of the pH to 4 or higher caused early agglomeration of the gelatin, which appeared as a solid gel, when acetone was added. At pH 2.5, the protein chains are highly positively charged, where a decrease in the pH below the isoelectric point (IEP) of gelatin type B (which is 4.5-5) leads to an increase in the zeta potential (ζ) of gelatin by protonation of aspartic acid and glutamic acid (pKa of 4.3). The electrostatic repulsion prevents the polymer chains from uncontrolled agglomeration. When the pH was increased to 8-12, the formation of turbid yellowish white sticky suspension was observed **(Table S1).**

The results showed that pH 2.5 was the optimum pH for the NPs preparation. It has been claimed that creating enough charges on the gelatin surface causes NP to develop and prevents subsequent agglomeration [1]. The process of GNPs development involves a regulated precipitation, in which the force of precipitation caused by the loss of water molecules is regulated by intermolecular charge repulsions generated by the altered pH [2].

Table S2 shows the effect of the amount and concentration of aqueous GLA solution as a cross-linking agent on EE and the actual drug loading (DL) of BBR loaded GNPs. The appropriate amount of the cross-linker is sufficient to stabilize the particles, however, lower amounts cannot cross-link or form NP pellets and therefore, fail to encapsulate the drug [3]. This can be ascribed to the gelatin swelling in aqueous media after the removal of organic solvent. Consequently, the results show that higher amount of the cross linker (185 μL) of 25 % v/v, longer crosslinking times 16 h and stirring speed 1250 rpm are associated with stable homogenous NP with low PDI, smallest PS, maximum EE 72.48 % ± 3.27 and DL 10.62 % ± 2.33 of the loaded GNPs. This can be ascribed to the fact that increasing the GLA amount leads to more entanglement of the particles by more interaction with the $–NH_2$ groups of

gelatin and prevents the particles swelling in water to increase stability and hardening of gelatin nanospheres [4].

Table S2. Effect of concentration and amount of glutaraldehyde (GLA) on encapsulation efficiency (EE) % and drug loading (DL) % of BBR – GNPs

Concentration of aqueous GLA solution (%)	Amount of GLA (µL)	EE (%) ± SD	DL (%) ± SD
**8**	**200**	—	—
**10**	**200**	**53.40 ± 8.89**	**7.83 ± 1.72**
**25**	**75**	**40.23 ± 8.07**	**1.50 ± 0.33**
**25**	**185**	**72.48 ± 3.27**	**10.62 2.33**

⁎Values represent mean ± Standard Deviation (SD) (n = 5).

Solubility Studies

A solubility study was performed to select the most appropriate dissolution medium that can provide sink conditions during the *in-vitro* release study [5]. Excess BBR (10 mg) was added to eight dissolution media (5 mL) prepared of phosphate buffers (PBS): pH (5.5, 7.4), pH 5.5 + 0.5 % Tween 80 (T80), and pH (5.5, 7.4) + 1 % T80 and pH (5.5, 7.4) + 30 % methanol (MeOH). Solutions were shaken in a shaking water bath at 37 ^{0}C and 200 rpm for 24 h, then incubated at 37 ^{0}C without shaking for another 24 h. Following 48 h of incubation, samples from each supernatant were filtered and diluted. The absorbance was measured at 344 nm to determine the concentration of the dissolved BBR base.

Figure S1 shows the solubility of unprocessed BBR powder in different dissolution media of PBS that were studied at 37 ^{0}C and 200 rpm. The findings clearly show that the solubility of BBR in PBS of pH 5.5 (0.83 mg/mL) is higher than that of pH 7.4 (0.61 mg/mL). It is observed that the addition of non-ionic surfactant as Tween 80 (T80) caused reduction in the solubility of BBR in both PBS of pH 5.5 and 7.4 (0.52 and 0.4 mg/mL respectively), due to that formation of non-ionizable complex of BBR salt and T80.

Interestingly, the addition of 30 % MeOH to PBS of pH 5.5 and 7.4 displays a significantly higher solubility of BBR (1.55 and 1.45 mg/mL respectively), where hydrogen bonding interactions are formed between MeOH and the electronegative oxygen atoms in BBR molecules.

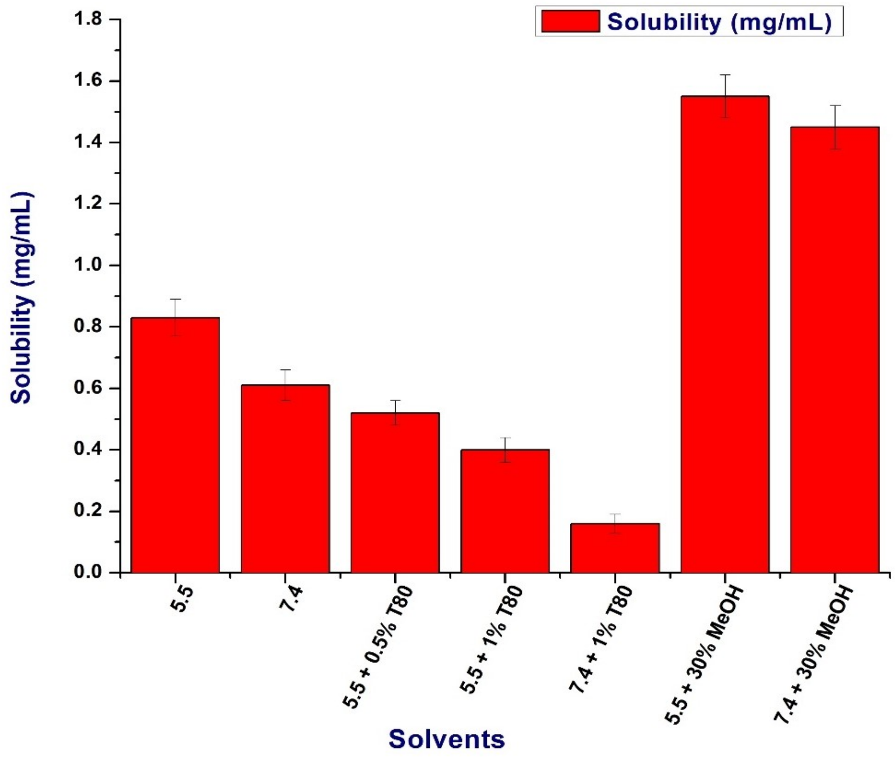

Fig. S1. Solubility studies of BBR in different dissolution media of PBS

Table S3. DPPH radical scavenging activity of plain GNPs 2 %, BBR-GNPs 2 %, and BBR-GNPs 5 %:

NP Concentration (µg/mL)	Inhibition ratio (%)		
	**plain GNPs 2 %**	**BBR-GNPs 2 %**	**BBR-GNPs 5 %**
**10**	1.84 ± 0.11	21.55 ± 0.31	34.07 ± 0.1
**20**	13.08 ± 0.09	28.91 ± 0.27	46.96 ± 0.11
**60**	22.10 ± 0.15	38.49 ± 0.26	53.04 ± 0.05
**80**	27.62 ± 0.5	49.36 ± 0.26	65.38 ± 0.05
**100**	35.73 ± 0.52	65.19 ± 0.15	92.08 ± 0.17
**DPPH IC50**	139.95 ± 0.48	81.04 ± 0.06	56.56 ± 0.05

***In-vitro* Hemolysis Assay**

A hemolysis test was performed to further assess hemocompatibility using rabbit Red Blood Cells (RBCs), which were collected in heparinized tubes following the method described by Farias *et al*. [6]. Plasma and buffy coat were removed by centrifugation (500 *xg* for 5 min) (SL 40 FR, Thermo Scientific, Germany) and RBCs were extensively washed twice with (150 mM) sodium chloride (NaCl) and PBS respectively. So, RBCs concentration in PBS solution is (0.5 % v/v).

First, a serial dilution of GNPs and BBR-GNPs was prepared in 0.9 % NaCl in range of (2-1000 µg/mL). Then, 1 % RBCs suspension (100 µL) was added to a new microtube containing (900 µL) of each compound previously diluted tested and incubated at 37 ^{0}C for 1 h. The tubes were centrifuged at 3000 *xg* for 5 min. The supernatant (200 µL) was placed in a 96-well plate and the absorbance was measured at 540 nm using a microplate reader. For positive (100 % hemolysis) and negative (0 % hemolysis) controls, the suspension of RBCs (100 µL) were mixed with 0.9 % NaCl (900 µL), respectively. The percentage of hemolysis was calculated as the following Eq. (7) [7]:

$$Hemolysis\,\% = \left[\frac{(Abs_{sample} - Abs_{C-})}{(Abs_{C+} - Abs_{C-})}\right] * 100$$

where, Abs_{sample} is the absorbance of tested samples, Abs_{C+} is the absorbance of positive control (100 % hemolysis) and Abs_{C-} is the absorbance of negative control (0 % hemolysis). All samples were assayed in triplicates. Finally, the inhibitory concentration (IC50) was estimated in µg/mL.

The *in-vitro* hemolytic toxicity of BBR-loaded GNPs is compared with GNPs. The results of BBR-GNPs depict a 3.5 times reduction in hemolysis of RBCs compared to GNPs as shown in **Fig. S2**. Hence, **Table S4** demonstrates that IC50 (µg/mL) of BBR-GNPs formula is higher than that of GNPs, which means that hemolytic activity is concentration-dependent. Therefore, as IC50 increases, so the nanoparticulate formula (BBR-GNPs) has good biocompatibility in hemostatic application [8]. As reported in literature [9], increasing the concentration of BBR in composite of biomimetic microspheres revealed the minimum hemolysis ratio in comparison with other formulations.

Table S4. Hemolysis ratio and IC50 of plain GNPs and BBR-GNPs:

Concentration (µg/mL)	Hemolysis ratio (%)	
	plain GNPs	BBR-GNPs
10	30.43	3.45
20	34.78	3.83
40	37.01	5.36

**60**	39.13	9.82
**80**	43.48	12.64
**100**	47.83	13.41
**IC50 (µg/mL)**	**104.55**	**316.36**

- IC50 (µg/mL): Extracts concentration that causes 50 % hemolysis of RBCs.

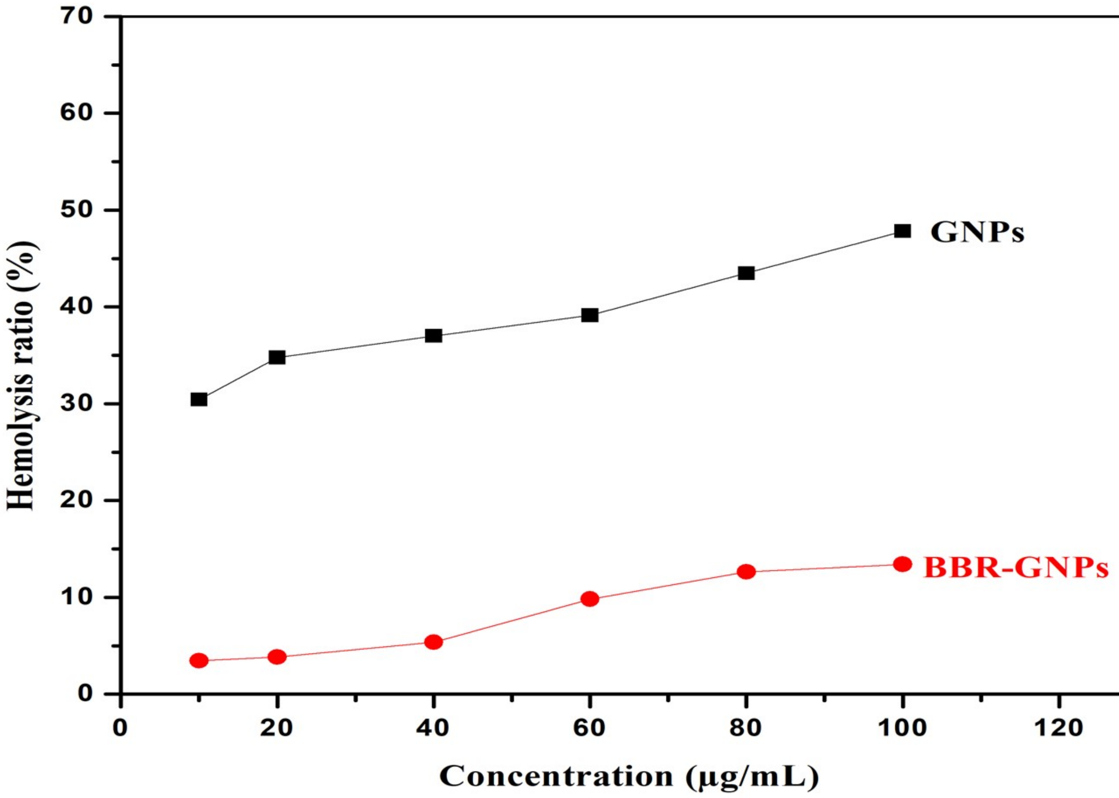

Fig. S2. Effect of different concentrations of GNPs and BBR-GNPs on hemolysis ratio (%)

References

[1] S. Azarmi, Y. Huang, H. Chen, S. McQuarrie, D. Abrams, W. Roa, W.H. Finlay, G.G. Miller, R. Löbenberg, Optimization of a two-step desolvation method for preparing gelatin nanoparticles and cell uptake studies in 143B osteosarcoma cancer cells, Journal of Pharmacy and Pharmaceutical Sciences 9 (2006) 124–132. https://doi.org/10.7939/R3J96097M.

[2] S.M. Ahsan, C.M. Rao, The role of surface charge in the desolvation process of gelatin: Implications in nanoparticle synthesis and modulation of drug release, Int J Nanomedicine 12 (2017) 795–808. https://doi.org/10.2147/IJN.S124938.

[3] Z. Babaei, M. Jahanshahi, M. Sanati, Fabrication and evaluation of gelatin nanoparticles for delivering of anti - cancer drug, International Journal of Nanoscience and Nanotechnology 4 (2008) 23–30.

[4] M. Shokry, R.M. Hathout, S. Mansour, Exploring gelatin nanoparticles as novel nanocarriers for Timolol Maleate: Augmented in-vivo efficacy and safe histological profile, Int J Pharm 545 (2018) 229–239. https://doi.org/10.1016/j.ijpharm.2018.04.059.

[5] M.U.K. Sahibzada, A. Sadiq, H. Sfaidah, M. Khurram, M.U. Amin, A. Haseeb, M. Kakar, Berberine nanoparticles with enhanced in vitro bioavailability: Characterization and antimicrobial activity, Drug Des Devel Ther 12 (2018) 303–312. https://doi.org/10.2147/DDDT.S156123.

[6] D.F. Farias, T.M. Souza, M.P. Viana, B.M. Soares, A.P. Cunha, I.M. Vasconcelos, N.M.P.S. Ricardo, P.M.P. Ferreira, V.M.M.I. Melo, A.F.U. Carvalho, Antibacterial, antioxidant, and anticholinesterase activities of plant seed extracts from Brazilian semiarid region, Biomed Res Int 2013 (2013). https://doi.org/10.1155/2013/510736.

[7] M.A. Mahmoud, D.A. Ghareeb, H.A. Sahyoun, A.A. Elshehawy, M.M. Elsayed, In Vivo Interrelationship between Insulin Resistance and Interferon Gamma Production: Protective and Therapeutic Effect of Berberine, Evidence-Based Complementary and Alternative Medicine 2016 (2016). https://doi.org/10.1155/2016/2039897.

[8] D.A. Ghareeb, S.R. Saleh, M.G. Seadawy, M.S. Nofal, S.A. Abdulmalek, S.F. Hassan, S.M. Khedr, M.G. AbdElwahab, A.A. Sobhy, A. saber A. Abdel-Hamid, A.M. Yassin, A.A.A. Elmoneam, A.A. Masoud, M.M.Y. Kaddah, S.A. El-Zahaby, A.M. Al-mahallawi, A.M. El-Gharbawy, A. Zaki, I. k. Seif, M.Y. Kenawy, M. Amin, K. Amer, M.A. El Demellawy, Nanoparticles of ZnO/Berberine complex contract COVID-19 and respiratory co-bacterial infection in addition to elimination of hydroxychloroquine toxicity, J Pharm Investig 51 (2021) 735–757. https://doi.org/10.1007/S40005-021-00544-W/TABLES/8.

[9] X. Zhang, K. Dai, C. Liu, H. Hu, F. Luo, Q. Qi, L. Wang, F. Ye, J. Jin, J. Tang, F. Yang, Berberine-coated biomimetic composite microspheres for simultaneously hemostatic and antibacterial performance, Polymers (Basel) 13 (2021) 1–16. https://doi.org/10.3390/polym13030360.
